# Novel Blotting Method for Mass Spectrometry Imaging of Metabolites in Strawberry Fruit by Desorption/Ionization Using Through Hole Alumina Membrane

**DOI:** 10.3390/foods9040408

**Published:** 2020-04-01

**Authors:** Hirofumi Enomoto, Masahiro Kotani, Takayuki Ohmura

**Affiliations:** 1 Department of Biosciences, Faculty of Science and Engineering, Teikyo University, Utsunomiya 320-8551, Japan; 2 Division of Integrated Science and Engineering, Graduate School of Science and Engineering, Teikyo University, Utsunomiya 320-8551, Japan; 3 Advanced Instrumental Analysis Center, Teikyo University, Utsunomiya 320-8551, Japan; 4 Hamamatsu Photonics K.K., 314-5 Shimokanzo, Iwata 438-0193, Japan; masa-kotani@etd.hpk.co.jp (M.K.); ohmura@etd.hpk.co.jp (T.O.)

**Keywords:** strawberry, metabolites, mass spectrometry imaging, desorption ionization using through hole alumina membrane (DIUTHAME), blotting

## Abstract

Mass spectrometry imaging (MSI) using matrix-assisted laser desorption/ionization (MALDI) is a powerful technique for visualizing metabolites in the strawberry fruit. During sample preparation for MALDI-MSI, sectioning of the samples is usually required. In general, MALDI-MSI analysis of strawberry fruits that are larger than a single glass slide is difficult because thin sections cannot be prepared. In this study, we attempted to visualize metabolites in large strawberry fruits by MSI, employing a blotting method that uses desorption ionization using a through-hole alumina membrane (DIUTHAME) chip. Large strawberry fruits were cut and a DIUTHAME chip was set on the cross-section to blot the metabolites. After drying the DIUTHAME chip, the metabolites were measured in positive and negative ion modes using a commercial MALDI-type mass spectrometer. Several peaks were detected in both the ion modes. Various metabolites related to food quality, such as sugars, organic acids, and anthocyanins, were detected and successfully visualized by blotting on a DIUTHAME chip in MSI. These results suggest that blotting using a DIUTHAME chip in MSI is useful for visualizing the metabolites present in the strawberry fruit.

## 1. Introduction

Various separation techniques, such as liquid chromatography-electrospray ionization (ESI)-mass spectrometry, are commonly used for qualitative and quantitative analyses of metabolites [1]. They are also used to investigate the analyte distribution by dividing samples into different tissues; however, the spatial resolution depends on the fineness of tissue differentiation. To overcome this problem, mass spectrometry imaging (MSI) has emerged for simultaneously investigating the spatial distribution of metabolites at a microscopic resolution without the need for probes such as antibodies [2,3,4,5,6,7,8]. MSI has been used to separate various metabolites based on their mass-to-charge ratio (*m/z*) and to visualize their distribution in tissues. Recently, MSI was adapted for analysis of metabolites in foods and was successfully used for visualization using soft ionization techniques, such as matrix-assisted laser desorption/ionization (MALDI) [9,10,11,12,13,14,15,16,17] or desorption ESI [18,19]. These techniques generally require preparation of thin sections (usually 10–20 μm) of the samples using a cryomicrotome.

Strawberry (*Fragaria* x *ananassa* Duch.) belongs to the family *Rosaceae*. It is a hybrid species of the genus *Fragaria*, bred in Europe in the 18th century as a cross between *F. chiloensis* (endemic to Chile) and *F. virginiana* (native to the eastern United States). Strawberry is currently widely cultivated and consumed worldwide because of its attractive appearance, good taste, and nutritional and health-promoting properties, making it an economically important fruit [20]. Strawberry is rich in nutritious metabolites, including sugars, vitamins, and organic acids, as well as in non-nutritious phytochemicals, particularly polyphenols [20,21]. Several studies have shown that polyphenols have antioxidative activities; thus, they are thought to be helpful in preventing chronic diseases [21]. The contents and balance of sweet metabolites, namely sugars, and sour metabolites, namely organic acids, are important factors defining the taste of the strawberry fruit. The predominant sugars are glucose, fructose, and sucrose, whereas the predominant organic acids are citric and malic acids [20]. In our previous study, major polyphenols, namely anthocyanins, such as pelargonidin-glycosides and flavan-3-ols, or sugars, and organic acids were visualized, using MALDI-MSI, in whole cross sections of strawberry fruit [14,15]. However, strawberry fruits larger in size than a single glass slide could not be analyzed because thin sections could not be prepared from the crumbling and fracturing tissues. Large strawberries are commonly sold as a high-grade fruit; therefore, an analytical method using MSI for larger strawberry fruits is required for its quality analysis.

Recently, we developed a novel ionization method, desorption ionization using through-hole alumina membrane (DIUTHAME) [22], which was adapted for MSI [23]. In the workflow, an area of hole porous alumina membrane in the DIUTHAME chip was set on the sample section attached to a conductive glass slide, and the surface of the DIUTHAME chip was measured in two dimensions using a commercially available MALDI mass spectrometer. Therefore, the DIUTHAME chip functions as a matrix in MALDI-MSI. Unlike MALDI, DIUTHAME is a matrix-free ionization method, and thus, the interferences caused by the matrix in the low mass range usually observed in MALDI are not observed in the DIUTHAME method [22,23]. Using this method, phospholipids in mouse brain sections were successfully visualized with high reproducibility and spatial resolution [23].

Blotting is one of the sample preparation methods that is not widely used for MSI. In this blotting method, metabolites are imprinted on the substrate; therefore, preparation of tissue sections is not required. A thin-layer chromatography plate, or commercially available MALDI target plate covered with cationic silver nanoparticles, was used as a substrate to visualize metabolites in strawberry fruit by desorption ESI- [24] or LDI-MSI [25], respectively.

In the present study, we visualized metabolites in strawberry fruits larger than a single glass slide using the DIUTHAME chip as a blotting substrate in MSI.

## 2. Materials and Methods 

### 2.1. Reagents

Methanol and water were purchased from Wako Chemicals (Tokyo, Japan). α-Cyano-4-hydroxycinnamic acid (CHCA) was purchased from Tokyo Kasei Co. (Tokyo, Japan). The peptide calibration standard, bradykinin (1–7) was purchased from Bruker (Billerica, MA, USA). All reagents and solvents used in this study were of analytical grade.

### 2.2. Strawberry Fruit

Plants of ‘Skyberry’ strawberry, one of the main cultivars in Japan, were cultivated in the Strawberry Research Center (Tochigi, Japan). Ten ripe strawberry fruits were harvested and those with cross-sectional sizes larger than a single glass side were used.

### 2.3. Preparation of DIUTHAME Chip

The DIUTHAME chip was prepared as described previously [22], with minor modifications. Briefly, the aluminum substrate was processed by wet anodization to through-hole porous alumina membranes approximately 20 μm in thickness. After bonding the membrane to a nickel-iron (Ni-Fe) alloy frame by vacuum-compatible epoxide adhesive, the device was dipped in 1.7 M phosphoric acid solution until the through-hole diameter and the open aperture ratio reached the desired values of 200 nm and 50%, respectively. Finally, platinum (Pt) was coated at 20 nm by electron beam evaporation only on the laser irradiation side. The DIUTHAME chip can be fabricated in various sizes according to the effective area of the through-hole alumina membrane and aperture geometry of the Ni-Fe alloy frame. In the present study, the chip size was same as a large glass slide (50 × 75 mm) and had an effective area of 40 × 65 mm.

### 2.4. Sample Preparation

**Figure 1:**
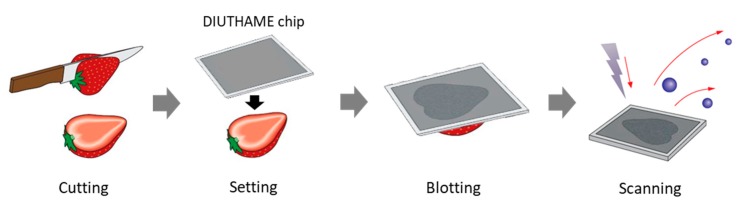
Workflow of the blotting method involving desorption ionization using through-hole alumina membrane (DIUTHAME) chip in mass spectrometry imaging.

The workflow is shown in Figure 1. A strawberry fruit was cut using a commercial knife. Immediately, the side of DIUTHAME chip not coated with Pt was placed on the cross-section of the strawberry and left for 5 min to blot the strawberry metabolites onto the chip. The chip was removed from the strawberry fruit, dried, and then used for MSI analysis.

### 2.5. MSI Analysis

MSI analysis was performed as described in our previous study [14], with some modifications. Briefly, the DIUTHAME chip was set to the MALDI target plate for the large glass slide (Bruker), and measured using an UltrafleXtreme (Bruker), which is a MALDI time-of-flight (TOF)/TOF mass spectrometer. Data were acquired using a step size of 300 μm in positive or negative ion modes and reflector mode. The laser diameter was set to the medium size. After measurement in the positive ion mode, different positions on the same chip were measured in the negative ion mode. The measured *m/z* ranges for both the ion modes were 100–800. The instrument was calibrated externally using the exact *m*/*z* values of CHCA [M + H]^+^ (*m/z* 190.04987) and bradykinin (1–7) [M + H]^+^ (*m/z* 757.39916) ions in the positive ion mode, or of CHCA [M − H]^−^ ions (*m*/*z* 188.03532) and bradykinin (1–7) [M − H]^−^ (*m*/*z* 755.38460) ions in the negative ion mode, as references on the DIUTHAME chip. The spectra were acquired automatically using the FlexImaging 4.1 software (Bruker). Mass spectra were normalized based on the total ion current using the same software. Two-dimensional ion-density maps were also created using the FlexImaging 4.1 software.

## 3. Results and Discussion

### 3.1. Blotting on DIUTHAME Chip in MSI Analysis

**Figure 2:**
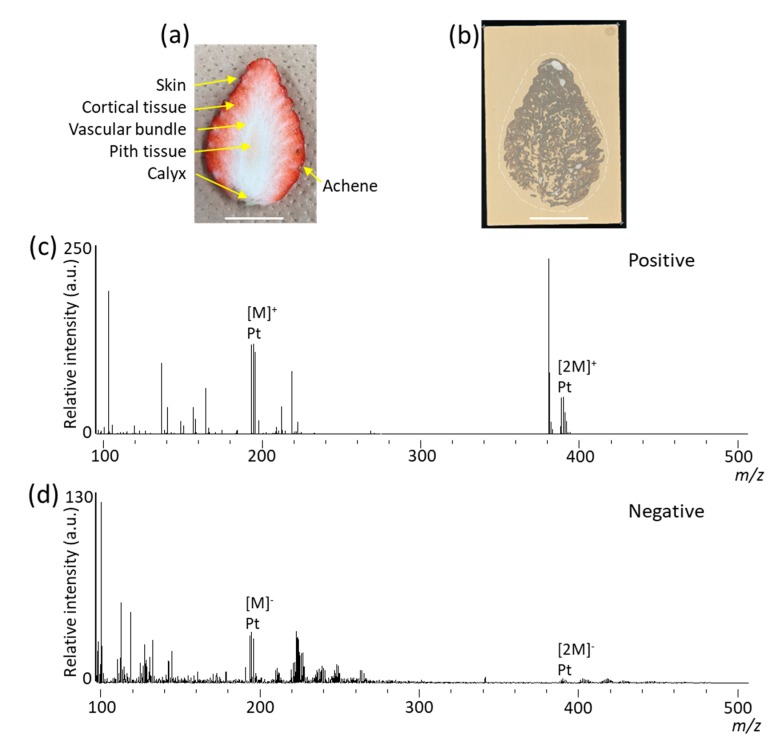
Mass spectra of strawberry fruit by blotting using desorption ionization using through-hole alumina membrane (DIUTHAME) chip in mass spectrometry imaging. (**a**) Optical image of the analyzed cross-section of strawberry fruit. (**b**) Optical image of DIUTHAME chip blotted with a cross-section of strawberry fruit. Dotted white line indicates the analyzed region. Scale bar = 20 mm. Mass spectra obtained using (**c**) positive and (**d**) negative ion modes. Peaks with platinum (Pt) are derived from Pt coated on the through porous hole alumina membrane.

Figure 1 shows the workflow used in the present study. We blotted metabolites onto the cross section of strawberry fruit on the DIUTHAME chip, which was used for metabolite analysis by MSI. Strawberry fruit larger than a single glass slide was used as the sample, as preparation of sections from large-sized fruit is generally difficult. In fact, we did try to prepare thin sections of large-sized strawberry fruit, freeze-embedded in carboxymethyl cellulose [14], using a cryomicrotome, by increasing the thickness to a maximum of 100 μm; however, sections retaining the original structures could not prepared. The optical image of the cross-section of strawberry fruit analyzed in this study is shown in Figure 2a. Six tissues, namely the skin, cortical tissue, vascular bundles, pith tissue, calyx, and achenes, were observed. The optical image of DIUTHAME chip after blotting of metabolites on the cross-section of strawberry fruit is shown in Figure 2b. MSI measurements obtained in positive and negative ion modes are shown in Figure 2c,d. Although the detection intensities appeared to be lower than those in MALDI-MSI analysis [14,15], numerous signal peaks were detected in the *m/z* range from 100 to 500. In the *m/z* range from 500 to 800, peaks were hardly detected. DIUTHAME is a matrix free ionization method different from MALDI [23], in which the detected peaks are usually derived from the metabolites present in the sample. These results suggested that various types of metabolites in strawberry fruit were detected in both ion modes using our blotting method with the DIUTHAME chip. This indicated that metabolites are extracted from a cross-section of strawberry fruit to the appressed DIUTHAME membrane via the moisture contained in the sample and are then transferred to the vicinity of the top surface of the DIUTHAME chip by capillary action of the through holes, finally being detected as gas-phase ions via surface-assisted laser desorption/ionization [22,23].

In the mass spectra obtained in positive ion mode, *m/z* 104.0, 219.0, 231.0, 271.0, and 381.1 were assigned as choline [M]^+^, hexose (glucose and/or fructose) [M + K]^+^, citric acid [M + K]^+^, pelargonidin [M]^+^, and sucrose [M + K]^+^ ions, whereas in the mass spectra obtained in negative ion mode, *m/z* 179.0, 301.0, and 341.1 were assigned as hexose (glucose and/or fructose) [M − H]^–^, quercetin and/or ellagic acid [M – H]^–^, and sucrose [M – H]^–^ ions based on previous studies [1,14,25,26] (Table 1). Peaks corresponding to cyanidin, another major anthocyanidin in strawberry fruit, and malic acid were not detected. A few peaks around *m/z* 195 and 390 observed in the mass spectra of both ion modes were also detected on the region where the cross-section of strawberry fruit was not blotted (Figure 2b and c), indicating that these peaks were derived from Pt coated on the hole porous alumina membrane. In our previous study using MALDI-MSI of ’Tochiotome‘ strawberry fruit, the peaks for pelargonidin-glycosides, such as pelargonidin-glucoside [M]^+^ ion (*m/z* 433.1), were detected to be stronger than those for pelargonidin [M]^+^ ion [14], whereas, in the present study, the peaks for pelargonidin-glycosides [M]^+^ ion could not be detected (Figure 2c). It is known that anthocyanidins are generally present in their sugar-bound forms, i.e. anthocyanidin-glycosides, which are referred to as anthocyanins, in plants. Detection of pelargonidin in our previous study [14] was considered to be caused by the detachment of sugar moieties from the pelargonidin-glycosides by in-source fragmentation [15], suggesting that the degree of in-source fragmentation of metabolites is higher in the DIUTHAME-MSI than in MALDI-MSI. DIUTHAME may be a harder ionization method than MALDI under the experimental conditions used in this study. 

### 3.2. Distribution of Metabolites in Strawberry Fruit

**Figure 3:**
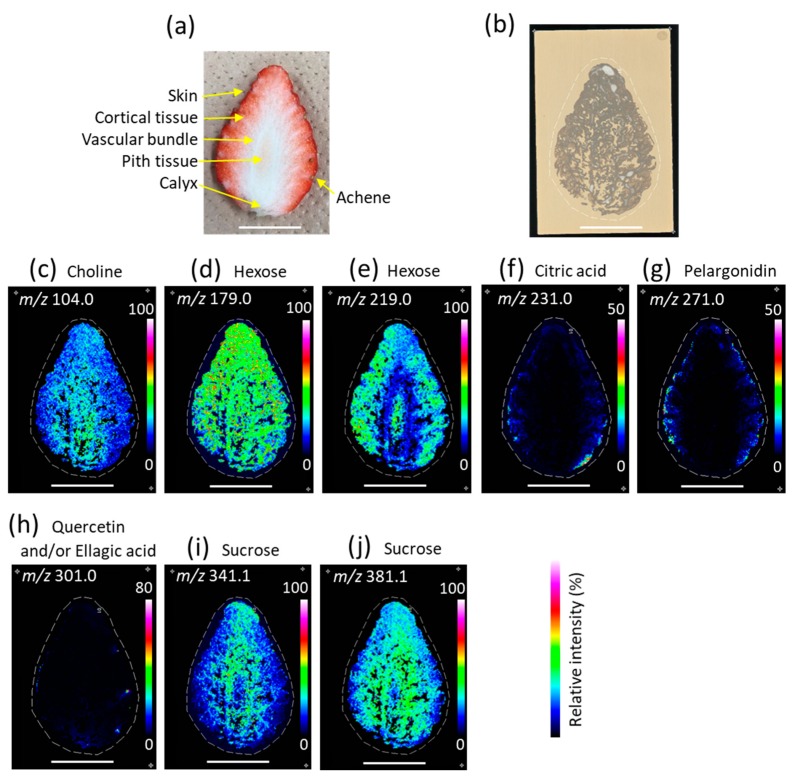
Visualization of the tentatively assigned metabolites in strawberry fruit. Optical images of (**a**) cross-section of strawberry fruit and (**b**) desorption ionization using through hole alumina membrane (DIUTHAME) chip blotted by the strawberry fruit. Ion images of *m/z* (**c**) 104.0, (**d**) 179.0, (**e**) 219.0, (**f**) 231.0, (**g**) 271.0, (**h**) 301.0, (**i**) 341.1, and (**j**) 381.1. Dotted white line indicates the analyzed region. Scale bar = 20 mm.

To investigate the distribution of the assigned metabolites in strawberry fruit, we reconstructed their ion images. Figure 3a,b show optical images of the cross section and blotted DIUTHAME chip, respectively. A vitamin like metabolite, choline was mainly distributed on the pith tissue (Figure 3c). In the negative ion mode, hexose was mainly distributed in the skin, cortical tissue, and pith tissue (Figure 3d), whereas, in the positive ion mode, hexose was almost equally distributed throughout the fruit (Figure 3e) as was observed in our previous study using MALDI-MSI [14]. Citric acid was predominantly distributed in the skin and a little of it was distributed in the cortical tissue (Figure 3f). Pelargonidin was mainly distributed in the skin and a little of it was distributed in the cortical tissue (Figure 3g). Quercetin and/ellagic acid were mainly distributed in the achene (Figure 3h). Ion images of sucrose observed using the positive and negative ion modes were similar; sucrose was mainly distributed in the vascular bundles and cortical tissue (Figure 3i,j).

In the present study, we visualized various metabolites in strawberry fruits larger than a single glass slide by blotting on the DIUTHAME chip in positive and negative ion modes of MSI. Pelargonidin-glycosides are orange/brick pigments in plants. The distribution of pelargonidin (Figure 3g) was similar to that of pigments in the cross-section of the strawberry fruit (Figure 3a), suggesting that the positional information of metabolites in the strawberry fruit was retained in the DIUTHAME chip during blotting. This result corresponded to that of phospholipid analysis in the mouse brain using the DIUTHAME chip in MSI [23]. However, the distribution patterns of hexose between positive and negative ion modes were clearly different. Interestingly, the distribution pattern of hexose in the positive ion mode was similar to that of red pigment observed in the cross-section of the strawberry fruit (Figure 3a, e). Hexoses were the major sugar moieties of pelargonidin-glycosides, and in the positive ion mode, vigorous in-source fragmentation was suggested for pelargonidin-glycosides (Figure 2c). Based on these results, the ion image of hexoses in the positive ion mode may be strongly affected by hexoses detached from the pelargonidin-glycoside.

In our previous analysis of strawberry fruit using MALDI-MSI, citric acid was found to be mainly distributed on the upper and lower sides of the cortical tissue [14], whereas, in the present study, it was mainly distributed in the skin and a little amount was also found in the cortical tissue (Figure 3f). This discrepancy might be due to the difference of the cultivar—‘Tochiotome’ was used for the MALDI-MSI analysis and ‘Skyberry’ was used in the present study—and/or due to the difference in the temperature of the fruit during cutting for sample preparations. In the MALDI-MSI analysis, frozen strawberry fruit was cut in a cryomicrotome camber (at approximately −20 °C), whereas, in the present study, raw strawberry fruit was cut at room temperature (approximately 20 °C). Enzymes present in strawberry fruit can perform their activity at room temperature. Therefore, distribution of citric acid may change as a result of enzymatic reactions during the period between cutting and blotting onto the DIUTHAME chip.

## 4. Conclusions

We demonstrated that blotting on the DIUTHAME chip in MSI is a useful method for visualizing metabolites in strawberry fruit, particularly in large-sized fruits. This method can be applied for metabolite analysis of other watery plant tissues other than the strawberry fruit. However, the detection intensities obtained by blotting using DIUTHAME were observed to be lower than those obtained by MALDI, and vigorous fragmentation occurred. Understanding the ion formation mechanism in DIUTHAME may be useful for overcoming this limitation. In addition, for the application of this blotting method using the DIUTHAME chip with a dried sample, an external solvent is required to extract metabolites. There were a lot of peaks that were not assigned. To identify the non-assigned metabolites, LC-MS analysis of metabolites extracted from the analyzed DIUTHAME chip or strawberry fruit is thought to be necessary. We expect that application of the blotting method using DIUTHAME-MSI will broaden, and could be used for the analysis of not only food samples but also various biological tissues by resolving these challenges.

## Figures and Tables

**Table 1:** Assigned metabolites on strawberry fruit cross-section.

m/z	Ion type	Metabolites ^1^
104.0	[M]^+^	Choline
179.0	[M − H]^–^	Hexose (glucose and/or fructose)
219.0	[M + K]^+^	Hexose (glucose and/or fructose)
231.0	[M + K]^+^	Citric acid
271.0	[M]^+^	Pelargonidin
301.0	[M − H]^–^	Quercetin and/or ellagic acid
341.1	[M − H]^–^	Sucrose
381.1	[M + K]^+^	Sucrose

^1^ Metabolites were assigned based on the *m/z* values and previously published literatures [1,14,25,26].
